# COVID-19 Presenting as Banti's Syndrome

**DOI:** 10.7759/cureus.9096

**Published:** 2020-07-09

**Authors:** Zohra R Malik, Zareen Razaq, Melody Siff, Saher Sheikh

**Affiliations:** 1 Internal Medicine, St. John's Episcopal Hospital, Far Rockaway, USA; 2 Internal Medicine, Ghurki Trust Teaching Hospital, Lahore, PAK; 3 Internal Medicine, Nassau University Medical Center, East Meadow, USA

**Keywords:** covid, banti syndrome, hematemesis, splenomegaly, portal hypertension, non-cirrhosis

## Abstract

COVID causing Banti's syndrome has not been reported in literature yet. Banti’s syndrome is a rare disorder characterized by splenomegaly, ascites, and portal hypertension without coexisting cirrhosis of the liver. Here we report a case of a 32-year-old man who presented with hematemesis, and further workup revealed that the patient had bleeding varices, ascites, and splenomegaly, thus completing the picture of Banti’s syndrome. Although this is a rare disorder, Banti's syndrome must be taken into account in a patient presenting with hematemesis and splenomegaly. The patient had flu-like symptoms for three weeks but did not seek any medical help and eventually presented with Banti's syndrome. His serology was positive for COVID-19. The coronavirus (COVID-19), discovered in 2019, has been creating havoc since it first emerged in China and is now spreading worldwide. Its presentation is somewhat similar to influenza.

## Introduction

Banti’s syndrome is characterized by raised portal venous pressure, causing portal hypertension due to either intrahepatic or pre-hepatic lesions but there is no liver pathology. Although it is a rare disease that occurs worldwide, it is more common in developing countries. Banti’s syndrome is known as hepatoportal sclerosis in the USA, non-cirrhotic portal fibrosis in Asia, idiopathic portal hypertension in Japan, and Banti’s syndrome in Europe [1]. It was first described by Guido Banti, an Italian physician in the year 1898 as a disease with splenomegaly, portal hypertension, and anemia with normal liver pathology [2,3]. The age of onset is usually third or fourth decade. Virus SARS-CoV-2 (severe acute respiratory syndrome coronavirus-2) or the 2019 novel coronavirus (2019-nCoV) belongs to the broad family of coronaviruses. There are four genera of coronaviruses: alpha, beta, gamma, and delta. The genera known to infect humans are alpha coronaviruses (the human coronavirus 229E (HCoV-229E), human coronavirus NL63 (HCoV-NL63), beta coronaviruses (human coronavirus HKU1 (HCoV-HKU1), human coronavirus OC43 (HCoV-OC43), the Middle East respiratory syndrome-related coronavirus (MERS-CoV), and the severe acute respiratory syndrome coronavirus (SARS-CoV) [4]. COVID-19 is a rapidly evolving virus. The aim of this case report is to establish an association between COVID and Banti's syndrome, which is a very rare and unusual finding in COVID-19 patients.

## Case presentation

**Figure 1:**
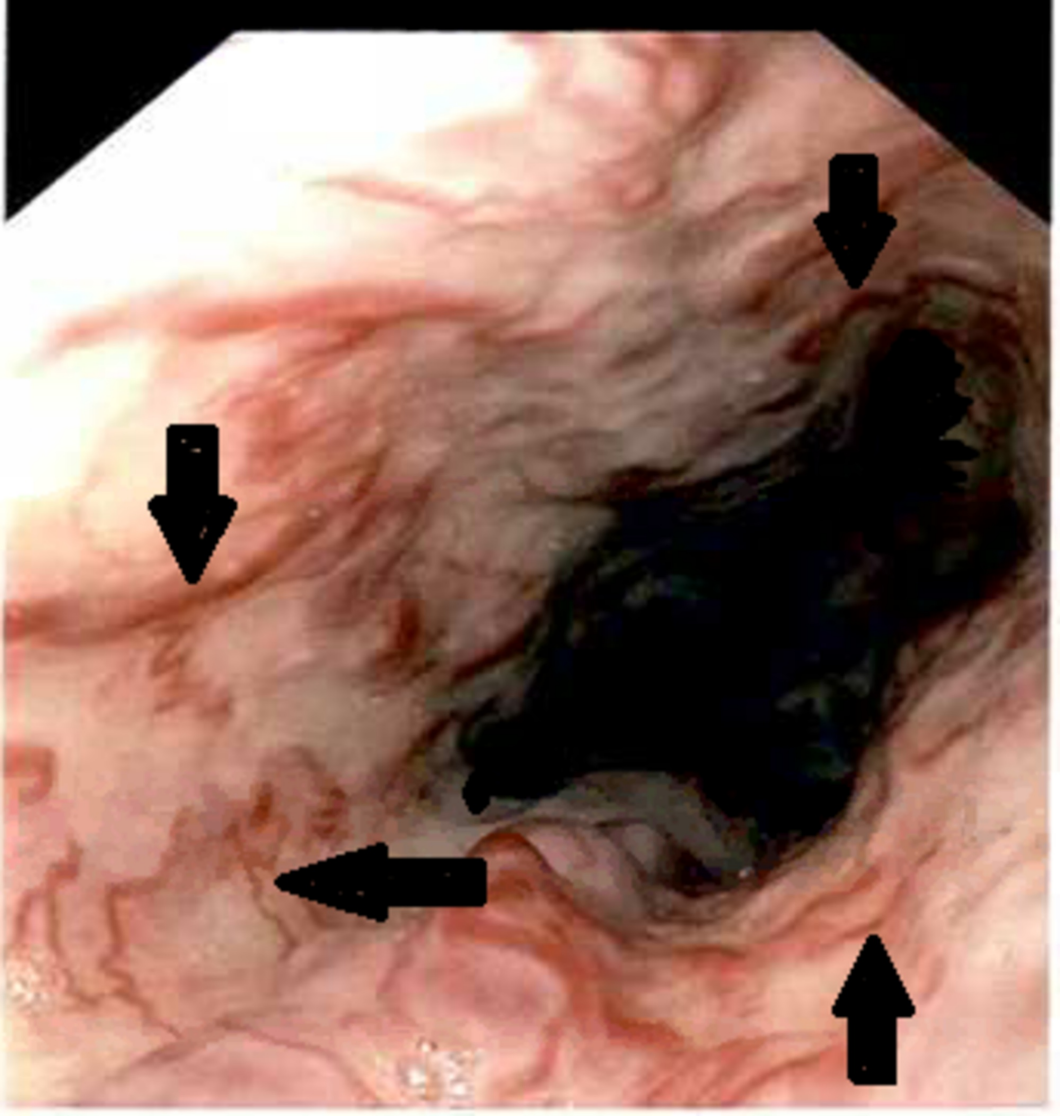
Endoscopic image showing multiple varices in the esophagus (black arrows)

We hereby discuss the case of a 32-year-old morbidly obese man (350 lbs), with a past medical history of hypothyroidism who presented at our hospital’s emergency department with an episode of hematemesis. The patient had a high-grade fever, rigors, and chills, non-productive cough, and left upper quadrant abdominal pain for the past two weeks for which he did not seek any medical help. The abdominal pain was 4/10 in intensity, located in the left upper quadrant, non-radiating, exacerbated after a meal. On the morning of the presentation, the patient vomited a cup full of dark red blood without any food particles. The fever and cough had resolved by the time of presentation. The patient denied shortness of breath, headache, bowel or bladder abnormalities, history of alcoholism, non-steroidal anti-inflammatory drug (NSAID) use, or liver disease. The patient had no known food or medication allergies. He was a lifetime non-smoker. There was no history of drug abuse or ethanol consumption. The patient took levothyroxine for 10 years. Uneventful appendectomy was done as a child. On examination, a morbidly obese man lying uncomfortably in bed. Blood pressure was 90/60 mmHg and pulse rate was 105 beats/minute. On inspection, his abdomen appeared distended. No scars or dilated veins were seen. The abdomen was tender to palpation in the left upper quadrant. The spleen was palpable. His cardiovascular, central nervous system, and respiratory system were all normal. A review of the peripheral smear revealed the presence of anisopoikilocytosis, large and giant platelets with some degree of clumping. White blood cell series reveals a predominance of neutrophilic activity with toxic granulation noted to the white blood cell series. His labs on presentation were as follows: hemoglobin 12.5 mg/dl (normal 12.0-18.0 g/dl) and hematocrit 39.5% (normal (42%-52%). The patient was given normal saline bolus which helped raise his blood pressure. His serology was positive for the COVID-19 virus. CT abdomen and pelvis showed fatty liver and splenomegaly, trace amounts of ascites, and minimal airspace disease in the right lower lobe. The transabdominal sonogram demonstrated a heterogeneously enlarged spleen measuring 17.1 cm. Hematologist and gastroenterologist were consulted, and the patient was prepared for emergency endoscopy. Two large-bore IV lines were placed, active type and screen was done, and the patient was placed nil per os (NPO). Protonix 40 mg IV Q12HR was started. The patient received a normal saline bolus. An upper endoscopy was done which showed multiple varices in the esophagus (Figure 1). Five esophageal bands were inserted to the varices prone to bleed. 

Octreotide was started. Hemoglobin significantly dropped from 12.5 to 4.8 mg/dl. The patient was transfused a total of six units of packed red blood cells (PRBCs), and two 200-mg bag of Venofer. Hemoglobin post-transfusion 7.8 mg/dl and hematocrit 23.8%. The patient felt better and wanted to leave. The patient was medically optimized and discharged with a follow-up GI appointment.

## Discussion

Patients with Banti's syndrome may present with a mass in the left upper quadrant due to splenomegaly or they may present with one or more episodes of gastrointestinal variceal bleeds resulting from portal hypertension. A number of hypotheses have been proposed, signifying limited understanding of the cause of Banti’s syndrome. An infection of the GI tract with repeated septic embolization of the portal circulation activating the stellate cells which cause fibrosis has been proposed as a possible etiology [5]. Injury predominantly manifests in the presinusoidal region. Other proposed causes include autoimmune disorders, chronic exposure to arsenic, prothrombotic states (e.g. factor V Leiden mutation), or copper sulfate (vineyard sprayers); prolonged treatment with methotrexate; hypervitaminosis A; and renal allograft recipients under treatment of 6-mercaptopurine, azathioprine, and corticosteroids [1]. However, the exact etiology in the majority of cases remains unknown. Anemia in these patients may be microcytic, hypochromic due to gastrointestinal blood loss or normocytic, normochromic due to hypersplenism [6,7]. COVID-19 is known to cause a hypercoagulable state but how it causes Banti's syndrome is still unknown. It could be through embolization of the portal circulation or directly causing thrombosis of the portal vessels. A lot of research needs to be done to find out the underlying mechanism leading to Banti's syndrome in a COVID-19 positive patient. 

## Conclusions

COVID-19 is an evolving virus and is known to cause a hypercoagulable state. Through this case report, we are trying to establish an association between COVID-19 and Banti's syndrome. A previously healthy person can develop Banti's syndrome if he is infected with COVID-19. If a person presents with hematemesis and splenomegaly as in our case report, it is appropriate to evaluate him for varices. There is a possibility of thrombus formation in the splenic vein leading to splenic vein thrombosis and portal hypertension and resulting varices. 
